# Measurement and decomposition of income-related inequality in self-rated health among the elderly in China

**DOI:** 10.1186/s12939-019-0909-2

**Published:** 2019-01-08

**Authors:** Hai Gu, Yun Kou, Hua You, Xinpeng Xu, Nichao Yang, Jing Liu, Xiyan Liu, Jinghong Gu, Xiaolu Li

**Affiliations:** 1 Center for Health Policy and Management Studies, Nanjing University, Nanjing, China; 2 Department of Social Medicine and Health Education, School of Public Health, Nanjing Medical University, Nanjing, China; 3 Nanjing Foreign Language School, Nanjing, China; 4 Department of Otolaryngology, The First Affiliated Hospital of Nanjing Medical University, Nanjing, China

**Keywords:** Corrected concentration index, Decomposition, Elderly, Income-related inequality, Self-rated health, China

## Abstract

**Background:**

Population ageing in China has brought increasing attention to the health inequalities of the elderly. The purpose of this paper is to measure income-related health inequality among the elderly in China and decompose its causes.

**Methods:**

The data are from the China Health and Retirement Longitudinal Study (CHARLS) survey in 2013, which contains 6176 individuals aged 60 years and above. A multiple linear regression model was used to analyze the influencing factors of self-rated health (SRH) among the elder people. Furthermore, the corrected concentration index were used to measure income-related health inequality. Wagstaff-type decomposition analysis was employed to explore the cause of inequality. The measurement and decomposition of health inequality was also performed separately in the male and female subgroups.

**Results:**

Most elderly declared their health status as “fair” (51.33%) or “poor” (21.88%). Income, gender, residence, region, health insurance and other factors had significant association with SRH (*P* < 0.05). The corrected concentration index (CCI) was 0.06, indicating pro-rich inequality in health among the elderly. Decomposition analyses revealed that the main contributors to health inequality included income, residence, region, health insurance, and employment. For female elderly, most of the inequality was due to residence (50.78%) and income (49.51%); for male elderly, most of the inequality was due to insurance (38.65%) and income (22.26%); for the total sample, employment had a negative contribution to health inequality (− 25.83%).

**Conclusion:**

The findings confirm a high proportion of elderly with poor SRH, and health inequality in the Chinese. Some socioeconomic strategies should be conducted to reduce this health inequality among the elderly, such as reducing income disparities, consolidating health insurance schemes, and narrowing urban-rural and regional gaps. Older females with low incomes in rural areas are a vulnerable subgroup and warrant targeted policy attention.

## Background

China is now becoming an aging society. By the end of 2014, the number of elders (aged 60 years and above) had reached 212 million, accounting for 15.5% of the population [1]*.* Moreover, it is estimated that by 2050, this will exceed 30% [2]. This increasing population of Chinese elderly faces emerging health challenges. Chronic diseases and disabilities have become more common. An earlier study shows that 33% of Chinese aged 60 years and older had chronic pain. Disability is also widespread, with 38% of participants reporting difficulties in daily living [3].

Owing to the huge population of the elderly, the socioeconomic burden caused by their health problems will be significant [4]. Aging conditions and health challenges will exert enormous pressure on pension, medical, and other related areas, and promote coping reforms in China. Therefore, it is necessary to examine the health status of the elderly Chinese.

Health assessment should include both the overall health levels and health inequality [5]. The measurement of inequalities in health requires information on health and socioeconomic status at the individual level. Some studies have used individual-level information to explore the extent of inequalities and the causal relationships between socioeconomic status and health inequality. A pan-European study indicated the presence of income-related health inequities across Europe, and that income inequality has a significant impact on health inequality [6]. Mangalore used a concentration index to measure income-related inequality in mental health in Britain and found marked inequality unfavorable to lower income groups [7]. A study using national data of Chinese elderly discovered that not only individual income affects their health but also provincial-level income does [8].

Meanwhile, many studies have further explored the causes of inequality besides income. Variables such as age, gender, education, employment, geographical location, medical insurance and health-related behaviors, have previously been identified as powerful sources of health inequalities [1, 6, 9–12]. Some determinants, such as age and gender are unavoidable, while others such as education, marital status, employment, personal behaviors, regional disparity, economic status, and medical insurance are avoidable. Health inequality due to avoidable factors generally is a form of health inequity, and it is necessary to eliminate or alleviate this through policy optimization.

Prior studies on elderly health have focused on health evaluation and factors associated with health outcomes and inequality. However, in terms of the determinants of health inequality in elderly, there is few quantitative analysis of influence extent. In addition, few studies have involved comparing health inequity and the causes in subgroups of older people, such as by gender. This study not only analyzes the health determinants, but also ascertains the contribution of each determinant to health inequality among the elderly, and even among the gender subgroups. The concentration index is a standard method to measure income related inequality in health [13]; furthermore, we can decompose it into contributions from impact factors [14]. Using data from the China Health and Retirement Longitudinal Study (CHARLS) survey in 2013, we calculated the corrected concentration index of self-rated health (SRH) to measure health inequality and employed a Wagstaff-type decomposition analysis [14] to explore the contribution of various characteristics to health inequality among Chinese elderly. For policy purposes, it is important to understand the relationships between the characteristics and health inequality of the elderly to target policies optimally. Further, we implemented the decompositions in gender subgroups to provide evidence for policy adjustments with gender differentiation.

## Methods

### Data and sampling

The data for this study are from the CHARLS survey in 2013, which is a national panel data set, conducted by the China Center for Economic Research of Peking University. CHARLS aimed to collect a set of high quality micro data that represents elderly individuals over 45 years old and their families in China. A multi-stage stratified random-sampling method was used to ensure that the samples were representative of the population [15]. The design of the study is similar to the English Longitudinal Study of Aging (ELSA), Survey of Health, Aging and Retirement in Europe (SHARE), and is available online [16].

The contents of this survey include the following aspects: 1. Demographic background, 2. Family information, 3. Health status and functioning, 4. Healthcare and insurance, 5. Work, retirement, and pension, 6. Income, expenditure, and assets. In 2013, the sample size was 18,605 individuals from 10,624 households. This study subjects were individuals 60 years or older, totaling 8957 individuals. After excluding cases with default values on related important items in this study (about 2700 individuals did not answer one or several questions about income, gender, residence, marriage, medical insurance, and so on), the final analyses included 6176 individuals (69.0%).

Ethical approval for this study was not necessary because it was based exclusively on publicly available data, CHARLS, and the study subjects were not directly approached.

### Dependent variable

In this study, the dependent variable indicates the self-rated health status of elderly. SRH is mainly based on the subjective judgments of the respondents themselves. In CHARLS, SRH is an ordinal categorical variable, and “1, 2, 3, 4, 5” represented “very good, good, fair, poor, very poor,” respectively. To facilitate interpretation, we re-encoded the variable; therefore, “1, 2, 3, 4, and 5” represent “very poor, poor, fair, good, and very good.”

### Independent variables

Independent variables were classified into four categories.

The first type describes the social demographic characteristics of individuals, including age, gender, educational level, marital status, employment, regions (western, central and eastern), and residence (rural and urban).

The second type describes the physical conditions of subjects. For example, the chronic diseases variable indicates whether the respondent has been diagnosed with a chronic disease. The CHARLS survey included 14 types of common chronic diseases. Similarly, the physical disabilities variable indicates whether the respondent has a listed physical disability. The CHARLS survey included five types of physical disabilities.

The third type of independent variable describes health-related behaviors and includes two dummy variables: smoke and medical examination. “Smoke” indicates whether the respondent has ever smoked or is a smoker, and “medical examination” indicates whether the respondent has undergone a medical examination in the last two years.

The fourth type of independent variable focuses on health insurance and economic conditions. According to the level of health insurance protection, we divide social health insurance into three categories. High-level health insurance includes urban employee basic medical insurance (UEBMI) and government medical insurance (GMI); middle level health insurance includes urban resident basic medical insurance (URBMI) and urban/rural resident basic medical insurance (URRBMI), and low-level health insurance includes the new rural cooperative medical system (NCMS). Few individuals in this study purchased commercial health insurance; therefore, the impact of commercial insurance has not been considered.

We measure the economic conditions of individuals with income. To reduce the impact of the economy scale of household income, we use equivalized per capita income (eqincome) to represent economic conditions, rather than actual per capita household income [17]. We calculate the equivalized per capita income as follows:

$$ \mathrm{eqincome}=\frac{household\ income}{{\left( family\ size\right)}^{0.56}} $$

### Statistical analysis

1) Regression Model.

We used multiple linear regression models to explore the factors influencing health. The model is as follows:

1 $$ {Health}_i=\alpha +\beta {X}_i+{\varepsilon}_i $$

where *X*_*i*_ is a matirx of independent variables, β stands for the vector of coefficients of independent variables, and *ε*_*i*_ is an error term.

2) Corrected concentration index.

The concentration index (CI) is a common measure of inequality in health [14]. The CI is twice the area between the concentration curve and the line of equality [18], calculated as follows:

2 $$ \mathrm{CI}=\frac{2}{n\mu}{\sum}_{i=1}^n{Health}_i{R}_i-1 $$

where *Health*_*i*_ is the health status of the i^th^ individual, μ is the mean of health variable, *R*_*i*_ is the fractional rank of the i^th^ individual in the income distribution. When the health variable is a bounded variable, the CI will estimate the extent of inequality incorrectly [19, 20]. To solve this problem, we adopted the corrected concentration index (CCI), developed by Guido [20]. The process of correcting CI is as follows:

3 $$ \mathrm{CCI}=\frac{4\mu }{\left(b-a\right)} CI $$

Where **μ** is the mean of the health variable, *b* is the upper bound and *a* is the lower bound. In this study, *b* equals 5 and *a* equals 1.

The value range of the CCI is (− 1, 1). When the CCI equals 0, health endowments are equally distributed between the poor and the rich. When the CCI is positive, health endowments are concentrated among the rich, and there exists pro-rich inequality. When the CCI is negative, health endowments are concentrated among the poor, and there exists pro-poor inequality. Larger absolute value of the CCI indicates more severe health inequality.

3) CCI Decomposition.

Using a Wagstaff-type [14] decomposition method to decompose the CCI, we can calculate the contribution of determinants to health inequality. The decomposition starts from the following regression:

4 $$ \frac{Health_i-a}{b-a}=\gamma +{\sum}_{j=1}^q{\lambda}_j{x}_{ji}+{\delta}_i $$

where *x*_1_, *x*_2_ … , *x*_*q*_ stand for the q independent variables, *λ*_1_, *λ*_2_ … , *λ*_*q*_ stand for the coefficients of independent variables, and *δ*_*i*_ represents the error term. Numerically, the coefficient *λ*_*j*_ in Eq. (4) equals *β*_*j*_ in Eq. (1) multiplied by $\frac{1}{b-a}$.

According to Eq. (4), we get:

5 $$ {Health}_i=\left(b-a\right)\left(\gamma +{\sum_j^q}_{=1}{\lambda}_j{\chi}_{ji}+{\delta}_i\right)+a $$

Substituting Eq. (5) and Eq. (2) into Eq. (3) yields:

6 $$ \mathrm{CCI}=4\left[{\sum}_{j=1}^q{\lambda}_j\overline{x_j}{CI}_j+\frac{2}{n}{\sum}_{i=1}^n{\delta}_i{R}_i\right] $$

where ${\overline{x}}_j$ is the means of determinant *x*_*j*_, *CI*_*j*_ is the CI of determinant *x*_*j*_. Eq. (6) shows that CCI can comprise of a deterministic component and residual component. The contribution of determinant *x*_*j*_ to total health inequality is $4{\lambda}_j\overline{x_j}{CI}_j$, and the contribution rate is $\frac{4{\lambda}_j\overline{x_j}{CI}_j}{CCI}\times 100\%$.

## Results

### Characteristics of the study population

Table 1 shows that the proportion of male elderly was 51.78%; average age was 67.6 ± 6.4 years old; proportion of rural elderly was 74.8%; proportion of physical disabilities was 31.9%, and smoke was 46.4%; proportion of chronic diseases was 73.6%, and medical examination was 45.5%. The mean of equivalized per capita income was 13.8 thousand Yuan.

**Table 1:** Description of variables (*N* = 6176)

	Total sample (N = 6176)		Female (*N* = 2978)		Male (*N* = 3198)	
Variables	N/ mean	%/SD	N/ mean	%/SD	N/ mean	%/SD
Age	67.63	6.36	67.45	6.42	67.81	6.31
Gender						
Female	2978	48.22				
Male	3198	51.78				
Educational level						
Illiteracy or Elementary	4847	78.48	2587	86.87	2260	70.67
Middle school and above	1329	21.52	391	13.13	938	29.33
Marital status						
Unmarried	1221	19.77	743	24.95	478	14.95
Married	4955	80.23	2235	75.05	2720	85.05
Employment						
Unemployed	2673	43.28	1441	48.39	1232	38.52
Employed	3503	56.72	1537	51.61	1966	61.48
Residence						
Urban	1554	25.16	657	22.06	897	28.05
Rural	4622	74.84	2321	77.94	2301	71.95
Regions						
Western	2156	34.91	1040	34.92	1116	34.90
Central	2093	33.89	1012	33.98	1081	33.80
Eastern	1927	31.20	926	31.09	1001	31.30
With chronic diseases						
No	1633	26.44	718	24.11	915	28.61
Yes	4543	73.56	2260	75.89	2283	71.39
With physical disabilities						
No	4207	68.12	2070	69.51	2137	66.82
Yes	1969	31.88	908	30.49	1061	33.18
Having ever smoked or smoking now						
No	3308	53.56	2641	88.68	667	20.86
Yes	2868	46.44	337	11.32	2531	79.14
Taking medical examination during the last two years						
No	3368	54.53	1624	54.53	1744	54.53
Yes	2808	45.47	1354	45.47	1454	45.47
Level of social medical insurance						
Low	4697	76.05	2374	79.72	2323	72.64
Middle	442	7.16	266	8.93	176	5.50
High	1037	16.79	338	11.35	699	21.86
Equivalized per capita income (thousand Yuan)	13.77	24.54	12.95	18.42	14.54	29.08
Self-rated health status						
Very poor	360	5.83	186	6.25	174	5.44
Poor	1351	21.88	731	24.55	620	19.39
Fair	3170	51.33	1505	50.54	1665	52.06
Good	835	13.52	379	12.73	456	14.26
Very good	460	7.45	177	5.94	283	8.85

The SRH of most elderly was “fair” or “poor.” The percentage of “fair” was 51.3, and 21.9% and 5.8% elder people declared their health status was “poor”, “very poor”, respectively.

### Factors associated with self-rated health

Table 2 presents the results of the multi-linear regression model. Elderly males had a higher SRH (*P* < 0.001) than elderly females. Elderly who had undergone medical examinations during the last two years had a lower SRH than others (*P* = 0.01). Furthermore, marital status had a significant impact on health (*P* = 0.03). The employed elderly were healthier than their unemployed counterparts were (*P* < 0.001). Moreover, the rural elderly had a lower SRH than the urban elderly (*P* = 0.003). Regional impact on elderly health was significant, with the following rankings: Eastern (*P* < 0.001), Central (*P* < 0.001) and Western. Chronic disease (*P* < 0.001) and disability (*P* < 0.001) showed significantly negative associations with elderly health. The level of social medical insurance had a significantly positive association with SRH (*P* < 0.05). In addition, the increase in equivalent per capita income improved the SRH of the elderly (*P* < 0.001).

**Table 2:** Factors influenced self-rated health of the elderly (Multiple linear regression model)

	Total sample (N = 6176)			Female (N = 2978)			Male (N = 3198)		
Variables	Coef. (*β*_*j*_)	95% Conf. interval	P	Coef. (*β*_*j*_)	95% Conf. interval	P	Coef. (*β*_*j*_)	95% Conf. interval	P
Age	0.003	(−0.001,0.007)	0.14	0.005	(−0.0009,0.01)	0.10	0.002	(−0.004,0.007)	0.56
Gender (Ref: female)	0.12^***^	(0.06,0.18)	< 0.001						
Educational level (Ref: Illiteracy or Elementary)	0.04	(−0.02,0.10)	0.20	0.06	(−0.05,0.17)	0.30	0.03	(− 0.05,0.11)	0.45
Marital status (Ref: unmarried)	−0.07^*^	(− 0.12,-0.01)	0.03	− 0.04	(− 0.12,0.04)	0.31	− 0.08	(− 0.17,0.007)	0.07
Employment (Ref: unemployed)	0.26^***^	(0.21,0.31)	< 0.001	0.21^***^	(0.14,0.28)	< 0.001	0.32^***^	(0.24,0.39)	< 0.001
Residence(Ref: urban)	−0.13^**^	(− 0.22,-0.04)	0.003	− 0.21^**^	(− 0.34,-0.08)	0.002	− 0.06	(− 0.19,0.06)	0.32
Regions (Ref: Western)									
Central	0.09^***^	(0.04,0.14)	< 0.001	0.11^**^	(0.04,0.19)	0.004	0.07	(−0.008,0.14)	0.08
Eastern	0.17^***^	(0.11,0.22)	< 0.001	0.17^***^	(0.09,0.25)	< 0.001	0.16^***^	(0.08,0.23)	< 0.001
With chronic diseases (Ref: no)	−0.43^***^	(− 0.48,-0.38)	< 0.001	− 0.40^***^	(− 0.48,-0.33)	< 0.001	− 0.46^***^	(− 0.53,-0.39)	< 0.001
With physical disabilities (Ref: no)	− 0.27^***^	(− 0.32,-0.22)	< 0.001	− 0.25^***^	(− 0.32,-0.18)	< 0.001	− 0.29^***^	(− 0.36,-0.22)	< 0.001
Having ever smoked or smoking now (Ref: no)	−0.06	(− 0.12,0.004)	0.07	− 0.04	(− 0.14,0.06)	0.47	− 0.07	(− 0.15,0.002)	0.06
Taking medical examination during the last two years (Ref: no)	− 0.06^**^	(− 0.11,-0.014)	0.01	− 0.06	(− 0.13,0.003)	0.06	− 0.06	(− 0.12,0.007)	0.08
Level of social medical insurance (Ref: low)									
Middle	0.10^*^	(0.001,0.21)	0.049	0.07	(−0.07,0.21)	0.32	0.11	(−0.05,0.26)	0.19
High	0.10^*^	(0.002,0.21)	0.045	0.007	(−0.16,0.17)	0.93	0.19^**^	(0.06,0.33)	0.005
Equivalized per capita income (thousand Yuan)	0.002^***^	(0.001,0.003)	< 0.001	0.003^**^	(0.0008,0.005)	0.007	0.002^**^	(0.0006,0.003)	0.004

^*^P < 0.05, ^**^P < 0.01, ^***^P < 0.001

The main determinants associated with SRH in the female group were similar to those in the male group, except for residence and medical insurance. Residence had statistical significance only in the female group, while medical insurance held such significance only in the male group (Table 2).

### Health inequality in elder population

Table 3 presents the health status of the elderly in different income groups. The SRH score for the poorest group was 2.81, while it was 3.02 for the richest group. High-income groups had higher SRH than low-income groups.

**Table 3:** Health status of different income groups and the inequality

	Different income groups	Self-rated health status(mean ± SD)	μ	CI	Corrected CI
Total	Quintile 1 (poorest)	2.81 ± 0.89	2.95	0.019	0.059
	Quintile 2 (poorer)	2.96 ± 0.93			
	Quintile 3 (middle)	2.97 ± 0.96			
	Quintile 4 (richer)	2.99 ± 0.98			
	Quintile 5 (richest)	3.02 ± 0.92			
Female	Quintile 1 (poorest)	2.85 ± 0.94	2.88	0.016	0.045
	Quintile 2 (poorer)	2.75 ± 0.91			
	Quintile 3 (middle)	2.83 ± 0.93			
	Quintile 4 (richer)	2.91 ± 0.90			
	Quintile 5 (richest)	3.03 ± 0.89			
Male	Quintile 1 (poorest)	2.85 ± 0.91	3.02	0.023	0.069
	Quintile 2 (poorer)	3.01 ± 0.96			
	Quintile 3 (middle)	3.04 ± 0.95			
	Quintile 4 (richer)	3.06 ± 0.96			
	Quintile 5 (richest)	3.13 ± 0.97			

For the total sample, the SRH score was 2.95, CI of SRH was 0.019, and the CCI was 0.06. For female elderly, the scores were 2.88, 0.016, and 0.045, respectively. For male elderly, the scores were 3.02, 0.023, and 0.069, respectively.

### Decomposition of health inequality

Table 4 shows the decomposition of health inequality. Avoidable social factors contributed 59.6% to health inequality. These avoidable social determinants mainly included income, residence, region, employment, and medical insurance. The contribution of income to health inequality was 28.2%; residence, 29.4%; employment, − 25.8%; medical insurance, 23.4%; and region, 5.5%.

**Table 4:** Inequality decompositions for CCI

Variables	Coefficients(*λ*_*j*_)	Means	Concentration Index	Contributions to CCI	Contributions (%)
Age	0.0007	67.63	− 0.002	− 0.0004	− 0.69
Male	0.030	0.52	0.017	0.001	1.86
Middle school and above	0.010	0.22	0.357	0.003	5.40
Married	−0.016	0.80	0.025	− 0.001	−2.25
Employed	0.065	0.57	−0.103	−0.015	−25.83
Rural	−0.033	0.75	−0.172	0.017	29.42
Regions					
Central	0.023	0.34	−0.038	− 0.001	−1.98
Eastern	0.042	0.31	0.083	0.004	7.44
With chronic diseases	−0.108	0.74	0.003	−0.0009	−1.57
With physical disabilities	−0.068	0.32	−0.083	0.007	12.42
Having ever smoked or smoking now	−0.014	0.46	−0.016	0.0004	0.71
Taking medical examination during the last two years	−0.015	0.46	0.080	−0.002	−3.71
Level of social medical insurance					
Middle	0.026	0.07	0.245	0.002	3.05
High	0.026	0.17	0.642	0.011	19.31
Equivalized per capita income (thousand Yuan)	0.0005	13.78	0.617	0.017	28.16
Main avoidable social factors(Income, residence, region, medical insurance, employment)				0.035	59.58
Total				0.042	71.75

Table 5 presents the decomposition of health inequality in gender subgroups. For female elderly, 87.0% inequality was attributable to avoidable factors and most of the inequality was due to residence (50.78%) and income (49.51%). For male elderly, 48.89% of the inequality was attributable to avoidable factors and most of the inequality was due to insurance (38.65%) and income (22.26%). In all, employment had a negative contribution to health inequality (− 25.83%).

**Table 5:** Inequality decompositions for CCI, by gender

	Female					Male				
Variables	Coefficients(*λ*_*j*_)	Means	Concentration Index	Contributions to CCI	Contributions (%)	Coefficients(*λ*_*j*_)	Means	Concentration Index	Contributions to CCI	Contributions (%)
Age	0.001	67.45	−0.004	−0.001	−2.42	0.0004	67.81	−0.001	−0.0001	− 0.15
Middle school and above	0.015	0.13	0.484	0.004	8.35	0.007	0.29	0.294	0.003	3.67
Married	−0.010	0.75	0.033	−0.001	−2.26	− 0.020	0.85	0.017	−0.001	−1.68
Employed	0.052	0.52	−0.098	− 0.011	−23.74	0.079	0.61	−0.109	− 0.021	−30.73
Rural	−0.052	0.78	−0.141	0.023	50.78	−0.016	0.72	−0.202	0.009	13.27
Regions										
Central	0.028	0.34	−0.036	− 0.001	−3.13	0.017	0.34	−0.039	− 0.0009	−1.29
Eastern	0.043	0.31	0.071	0.004	8.54	0.039	0.31	0.094	0.005	6.73
With chronic diseases	−0.101	0.76	0.0005	−0.0001	− 0.33	− 0.114	0.71	0.007	−0.002	−3.09
With physical disabilities	−0.063	0.30	−0.095	0.007	16.31	−0.072	0.33	−0.076	0.007	10.48
Having ever smoked or smoking now	−0.009	0.11	−0.088	0.0004	0.82	−0.019	0.79	−0.021	0.001	1.80
Taking medical examination during the last two years	−0.015	0.45	0.070	−0.002	−4.35	−0.014	0.45	0.089	−0.002	−3.38
Level of social medical insurance										
Middle	0.018	0.09	0.261	0.002	3.72	0.026	0.06	0.236	0.001	1.98
High	0.002	0.11	0.714	0.0006	1.32	0.048	0.22	0.597	0.025	36.67
Equivalized per capita income (thousand Yuan)	0.0007	12.95	0.613	0.022	49.51	0.0004	14.54	0.620	0.015	22.26
Main avoidable social factors(Income, residence, region, medical insurance, employment)				0.039	87.00				0.034	48.88
Total				0.046	103.12				0.039	56.54

## Discussion

This study found that the Chinese elderly are not very healthy. More than 25% of the elderly declared SRH as “poor” or “very poor,” more than 70% had chronic diseases, and more than 20% had physical disabilities. The Chinese elderly are less healthy than other Asian elder population. Healthy life expectancy (HALE) at age 60 was 15.8 in China, while 20.9 in Japan and 21.0 in Singapore [21]. China’s huge numbers of elderly and their increasing health problems will exert unprecedented pressure on healthy aging development. Meanwhile, the positive CCI showed that health endowments were concentrated among the rich, with pro-rich inequality in elderly health.

This study revealed that some social factors, including income, residence, region, medical insurance, not only influenced the health of the elderly, but also played critical roles in contributing to health inequality.

Income is the most important factor affecting the health inequality of the elderly. Prior studies in both developed and developing countries have come to a similar conclusion [22–24]. Income disparity creates differences in other health determinants, such as food consumption and the use of healthcare services [25]. A sufficient income provides the elderly with more opportunities for effective healthcare and entertainment. Although the Chinese healthcare system has undergone significant reforms, it is still difficult for low-income individuals to obtain necessary healthcare services [26]. This may be one reason why the elderly poor have lower SRH scores than rich ones. This indicates that it is possible to reduce health inequality among the elderly by reducing the income gap and improving the economic status of the elderly poor.

This study showed that residence has a distinct contribution to health inequality, and the rural elderly have lower levels of health than the urban elderly do. This is already clear evidence. An early paper [27] examined the Chinese healthcare system and urban-rural differences after the economic reforms in 1978. Differences in health outcomes between urban and rural population were apparent, and most adversely affected the rural elderly. Generally, the urban elderly have better opportunities to acquire public services. The study [27] also noted that urban-rural health disparities could be primarily due to wide variations in healthcare spending, resource allocation, and distribution of facilities and professionals. Another study [28] concluded that health inequality might be associated with discrepancy in access to basic public services between rural and urban areas, such as education, living conditions, and primary healthcare. However, the inequality of rural-urban residences among Chinese elderly continues to exist, despite the rapidly growing economy and the greatly improved conditions in many rural areas. Therefore, reducing the gap between the urban and rural elderly is a major task for the future.

Meanwhile, unbalanced socioeconomic development caused regional disparity to become an important factor affecting health inequality. This study demonstrated regional contribution to health inequality. The SRH of the elderly in the central and eastern regions is better than that of the elderly in the western regions. The findings were similar to a previous study [29], which revealed the gaps in health outcomes between developed coastal areas and most western provinces in China. With economic development and accelerated urbanization, large populations of labor in the central and western regions migrated to the eastern coastal areas, leaving significant numbers of elderly behind in the western rural areas [30]. These left-behind elderly usually do not possess sufficient financial resources, and such long-term separation from their children results in lack of care. They are the “empty-nesters,” and the health challenges of this group are a major challenge for healthy ageing in China.

This study noted that health insurance was strongly associated with health. The higher the medical insurance levels for the elderly, the higher the SRH. This outcome differs from an early study that reported no relationship between health insurance and SRH [31]. However, a prior finding similar to our study noted that health insurance does improve health status, as it enhances access to health services, especially for the elderly [32]. Meanwhile, our results confirmed the conclusion of a prior study, which found that China’s social health insurance also leads to health inequality when promoting health status [11]. In China, the fragmentation in social health insurance schemes generate inequity in accessing healthcare and financial protection for elderly covered under different schemes. Therefore, accelerating the consolidation of existing health insurance schemes is an urgent necessity, which will reduce disparities among different schemes in terms of fund levels and benefit packages.

In addition, this study also found a significant contribution of employment status in reducing health inequality. The employed are generally healthier [33] and employment provides many benefits, including income. For older workers, employment can provide opportunities to escape isolation and build self-worth [34]. The results showed that the proportion of employed elderly was larger in poor groups than in rich groups (the CI of employment was − 0.11). This may explain why employment contributed significantly in reducing pro-rich health inequality among the Chinese elderly.

Furthermore, the results revealed that both income and residence had a bigger contribution rate to health inequality among older women than it did in older men. Owing to historical and social reasons, a high proportion of elderly women engaged in years of unpaid housework with extremely low or no income. Especially in rural areas, most of the older women have no pension and retirement savings [35]. Economic status determines their inferior status in home and society, and significantly affects health outcomes. Hence, female elderly with low-income in rural areas are a subgroup that needs great attention by health policymakers. Additionally, we found that differences in insurance had a greater impact on health inequality in older men than in older women. Why was there a disparity between gender subgroups? Will there be any change after integrating medical insurance schemes? These questions deserve further exploration, particularly considering the reform of health insurance schemes in China.

This study also has several limitations. First, we used a subjective evaluation indicator— self-rated health—instead of objective indicators such as clinical examination results and prevalence of chronic diseases. Some bias may be evident because of the inadequate understanding of self-estimated health, especially among older respondents. Second, because of default values, we had to exclude 31% of the cases from the sample, which might result in some biases. Third, considering that an ordinal categorical scale measures the dependent variable, the ordered probit model seems more applicable. However, using the Wagstaff-type decomposition method to decompose inequality limits the form of the regression equation to a linear regression model [36]. Meanwhile, as the marginal effects calculated from the ordered probit model are close to the coefficients of OLS estimation [37], using the linear regression model is also acceptable. (Appendix: The results of ordered probit regression) Future studies should consider other appropriate decomposition methods based on a non-linear model, such as the ordered probit model and ordered logistic model.

## Conclusions

In summary, this study extends our knowledge of the effect of avoidable socio-economic determinants in income, residence, region, health insurance, and employment, on the self-reported health status of the Chinese elderly. These findings have significant policy implications. First, it is necessary to distribute more health resources to rural areas, particularly in the west to reduce gaps between different regions. Second, the government must accelerate the reform of medical insurance consolidation to reduce inequality due to fragmentation of insurance schemes. Third, the government should accelerate the implementation of poverty alleviation projects to reduce the proportion of the elderly poor.

A significant result of this study is that there are differences in the main factors that contribute to the health inequality in older men and women. In China’s aging society, the population of elderly women is larger than that of men [35]. The three disadvantages of gender, region, and age, make rural elderly women the most vulnerable group in Chinese society. The health problems of this subgroup need immediate attention and in-depth study.

## Appendix

**Table 6:** Factors influenced self-rated health of the elderly (Ordered probit regression model)

Variables	Coef.	95% Conf. interval	P
Age	0.003	(−0.001,0.008)	0.14
Gender (Ref: female)	0.14^***^	(0.07,0.22)	< 0.001
Educational level (Ref: Illiteracy or Elementary)	0.06	(−0.02,0.13)	0.20
Marital status (Ref: unmarried)	−0.08^*^	(−0.15,-0.01)	0.03
Employment (Ref: unemployed)	0.33^***^	(0.26,0.39)	< 0.001
Residence(Ref: urban)	−0.17^**^	(−0.27,-0.06)	0.003
Regions (Ref: Western)			
Central	0.11^***^	(0.04,0.17)	< 0.001
Eastern	0.20^***^	(0.13,0.27)	< 0.001
With chronic diseases (Ref: no)	−0.52^***^	(−0.59,-0.46)	< 0.001
With physical disabilities (Ref: no)	−0.34^***^	(−0.40,-0.28)	< 0.001
Having ever smoked or smoking now (Ref: no)	−0.07	(−0.14,0.01)	0.08
Taking medical examination during the last two years (Ref: no)	−0.07^**^	(−0.13,-0.02)	0.01
Level of social medical insurance (Ref: low)			
Middle	0.14^*^	(0.01,0.26)	0.04
High	0.14^*^	(0.02,0.27)	0.03
Equivalized per capita income (thousand Yuan)	0.002^***^	(0.001,0.004)	< 0.001

Model 2: Ordered probit regression model

^*^*P* < 0.05, ^**^*P* < 0.01, ^***^*P* < 0.001

## Abbreviations

CCI: Corrected Concentration Index
CHARLS: China Health and Retirement Longitudinal Study
CI: Concentration Index
HALE: Healthy life expectancy
SRH: Self-rated Health
